# Impact of a Multicomponent Digital Therapeutic Mobile App on Medication Adherence in Patients with Chronic Conditions: Retrospective Analysis

**DOI:** 10.2196/17834

**Published:** 2020-08-12

**Authors:** Elyssa Wiecek, Andrea Torres-Robles, Rachelle Louise Cutler, Shalom Isaac Benrimoj, Victoria Garcia-Cardenas

**Affiliations:** 1 Graduate School of Health University of Technology Sydney Sydney Australia; 2 Pharmaceutical Care Research Group University of Granada Granada Spain

**Keywords:** medication adherence, medication compliance, mobile phone, mobile apps, mHealth, gamification

## Abstract

**Background:**

Strategies to improve medication adherence are widespread in the literature; however, their impact is limited in real practice. Few patients persistently engage long-term to improve health outcomes, even when they are aware of the consequences of poor adherence. Despite the potential of mobile phone apps as a tool to manage medication adherence, there is still limited evidence of the impact of these innovative interventions. Real-world evidence can assist in minimizing this evidence gap.

**Objective:**

The objective of this study was to analyze the impact over time of a previously implemented digital therapeutic mobile app on medication adherence rates in adults with any chronic condition.

**Methods:**

A retrospective observational study was performed to assess the adherence rates of patients with any chronic condition using Perx Health, a digital therapeutic that uses multiple components within a mobile health app to improve medication adherence. These components include gamification, dosage reminders, incentives, educational components, and social community components. Adherence was measured through mobile direct observation of therapy (MDOT) over 3-month and 6-month time periods. Implementation adherence, defined as the percentage of doses in which the correct dose of a medication was taken, was assessed across the study periods, in addition to timing adherence or percentage of doses taken at the appropriate time (±1 hour). The Friedman test was used to compare differences in adherence rates over time.

**Results:**

We analyzed 243 and 130 patients who used the app for 3 months and 6 months, respectively. The average age of the 243 patients was 43.8 years (SD 15.5), and 156 (64.2%) were female. The most common medications prescribed were varenicline, rosuvastatin, and cholecalciferol. The median implementation adherence was 96.6% (IQR 82.1%-100%) over 3 months and 96.8% (IQR 87.1%-100%) over 6 months. Nonsignificant differences in adherence rates over time were observed in the 6-month analysis (Fr(2)=4.314, *P*=.505) and 3-month analysis (Fr(2)=0.635, *P*=.728). Similarly, the timing adherence analysis revealed stable trends with no significant changes over time.

**Conclusions:**

Retrospective analysis of users of a medication adherence management mobile app revealed a positive trend in maintaining optimal medication adherence over time. Mobile technology utilizing gamification, dosage reminders, incentives, education, and social community interventions appears to be a promising strategy to manage medication adherence in real practice.

## Introduction

Strategies to manage medication adherence, which is defined as the process by which patients take their medications as prescribed [1], are widespread in the literature and are reported to be modestly effective [2]. Most likely due to the multidimensional nature of medication-taking behavior and numerous determinants of nonadherence [3], multicomponent interventions with both technical and educational aspects have shown the most success [4]. However, these strategies have failed to find success in the real world; patient adherence levels tend to decrease in the long term and stay consistently at around 50% [5]. These strategies are limited not only by the capacity of the health care system delivering them but also by low levels of patient engagement. Even when patients are aware of the risks and consequences of diseases, few engage persistently in therapies to improve health outcomes [6-8].

Cognitive biases resulting in irrational and unhealthy behavior may be a key contributor to patient engagement in preventative health strategies. In contrast with traditional economic models of rational choice, modern insights have suggested that human behavior is highly influenced by the context or environment of our decision-making process rather than by price signals or factual information [9]. The field of behavioral economics combines psychology and neoclassical economics to shed light on the errors in mental processing that prevent patients from making rational and beneficial decisions to improve their health [10]. Some health behaviors may require high levels of self-control, meaning that a patient may need to endure “certain and immediate inconveniences in return for uncertain and distant benefits [11].” Obvious behaviors that create this paradigm are healthy food choices and exercise [12]. However, medication adherence, or the act of taking a medication at a certain time each day, creates inconvenience by disrupting the patient’s daily lifestyle or causing adverse effects; meanwhile, this behavior is only rewarded with uncertain and distant future health outcomes.

Strategies to influence cognitive biases include incentives and rewards. Incentives and rewards not only impact motivation but also create an immediate benefit to counteract inconvenience [13]. In previous literature, financial incentives showed success in improving medication adherence but were limited by long-term viability and capacity of resources, with economic incentives often eroding the potential economic gain [13-19]. The use of lottery-based incentives has also shown success in sustainment of adherence and long-term engagement [20,21]. Frequent lotteries with small rewards can engage patients based on regret aversion, namely the understanding that the emotional cost of regret (ie, missing a reward by not taking a medication dose) is significant [21].

Methods of gamification or use of nonfinancial extrinsic motivators, such as accruing “points,” can be feasible and practical ways to create similar senses of gratification and motivation [22]. Gamification is the application of game elements for purposes other than their expected use for entertainment [23]. An individual’s choice to engage in an activity is affected by extrinsic and intrinsic motivation. Medication adherence requires intrinsic motivation driven by internal rewards; this sense of motivation can often be difficult to achieve for behavior that has uncertain and distant health benefits. Through the use of gamification, extrinsic motivators such as earning points and monetary rewards can create and trigger internal motivation [23]. Gamification is not only able to use both extrinsic and intrinsic motivation to create consistent engagement through rewards, such as points or daily streaks, but can also create a sense of achievement [23]. Both gamification and rewards appear to be promising strategies to potentiate the effects of frequently used adherence management approaches, such as educational components and reminders.

Currently, over 300,000 mobile health (mHealth) apps are available; they have become common and instrumental tools for health behavior change in modern times [24,25]. Success has already been demonstrated with using mobile phone apps to support health behavior changes, ranging from constructing a healthy diet to managing chronic pain or improving physical activity [26-28]. Despite the potential of mobile phone apps as a tool to manage medication adherence, there is limited evidence of the effectiveness of these innovative interventions [29-31]. Real-world evidence, which refers to health care information gathered outside clinical research settings, can help minimize this evidence gap. Generated through the analysis of multiple sources, including electronic health records and mHealth apps, real-world evidence can be used to test how health interventions work in usual practice [32]. Observational studies of real-world data can assist in evaluating the potential impact of implemented health interventions in real world settings, such as interventions delivered through mobile phone apps [33].

The objective of this study was to use real-world data to analyze the impact over time of a previously implemented digital therapeutic mobile app on medication adherence rates in adults with any chronic condition. The impact on timing adherence rates was also analyzed.

## Methods

### Study Design

This was a retrospective observational study using real-world data. The implementation adherence of people in Australia using a commercially available smartphone application, Perx, was evaluated. The ESPACOMP Medication Adherence Reporting Guideline (EMERGE) and STrengthening the Reporting of OBservational Studies in Epidemiology (STROBE) Statement were used [1,34].

### Intervention: Perx Digital Therapeutic

Perx is a digital therapeutic that uses different components within a mobile app to improve adherence to medications. These include technical components (through dosage reminders based on the individual patient’s dosing regimen and individualized visual adherence feedback), educational components (through the use of educational materials on the disease and medications used), incentives and rewards (lottery-style delivery of gift cards), a social community (through a chat forum and collaborative competition dynamics), and gamification (through the use of point-earning and minigames to enhance the medication-taking experience). Perx enables users to input their medication schedule information while sending dosage reminders based on the individual patient’s regimen. Doses taken are self-reported and recorded by mobile direct observation of therapy (MDOT) photo verification [35]. “Gold” points are rewarded to users for each dose taken on time (±1 hour). Additionally, different minigames are offered at the time of a medication dose to enhance the medication-taking experience. The patient can earn extra gold points through learning a daily fact about their medication or disease state and by completing all daily tasks. Supplementary tasks within the app include health measurements, appointment reminders, physical therapy sessions, and other health actions, which provide users with a comprehensive system to track their health in addition to visual adherence feedback on their personal progress. A social forum and leaderboard component are also included, which create a Perx community. Reward shopping vouchers for popular stores can be redeemed either with a certain amount of gold earned or randomly by taking a correct dose. Screenshots showing the different features of the app can be found in Multimedia Appendix 1.

### Data Source and Patients

Deidentified user data from the Perx database were analyzed for this study to assess adherence dosing data between October 2018 and May 2019 within Australia. All information was deidentified, including medications, doses, schedules, user age, dosages taken and missed, and timestamps of dosages taken.

Users were recruited to use the app via a range of channels, including patient advocacy organizations (ie, Cystic Fibrosis Australia and Diabetes NSW & ACT), local community pharmacies, outpatient clinics at local hospitals, and app stores. App users with any chronic condition were included in the analysis. Two user cohorts were analyzed: one for users who used the app consistently for over 6 months and one for users who used the app consistently for 3 months. Users were excluded from the analysis if they used the intervention for less than 30% of the time period defined by the number of days active on the app. The 30% threshold was used because it excluded patients who appeared to decide to stop using the app during the time period of the analysis, as the objective was to analyze user medication adherence rather than adherence to the app itself.

A subanalysis of timing adherence was also performed for both time periods. Users were excluded from the subanalysis if timestamps were not available for the entire time period.

### Outcome: Medication Adherence

Adherence implementation rates (where adherence implementation was defined as the extent to which a patient’s actual dosing corresponded to the prescribed dosing regimen [1]) were calculated by dividing doses taken by total doses scheduled per 30-day period. This included doses taken outside the ±1-hour time period and was verified by comparing the recorded timestamps to the dosing schedules inputted within the app.

For the subanalysis, timing adherence was assessed with doses taken at the correct time (±1 hour) over total doses scheduled per 30-day period. This additional analysis was performed to understand the effects of the incentives, as users could only redeem incentives if the medication was taken within the ±1 hour time threshold. Both adherence measures are presented as percentages. Rates were compared to an optimal adherence level of 80%, which is the most commonly used cutoff point in the literature [36,37].

### Data Analysis

Data were analyzed by integrating the PROC SQL (SAS University Edition 9.4) and Python (Jupyter Lab 1.0) language programs and Microsoft Excel 2019 (Microsoft Corporation) to organize and retrieve the results. The analysis was conducted in 30-day time periods. Study variables were summarized using mean (SD) and median (IQR). Adherence variables were verified for normal distribution using the Shapiro-Wilk test. Due to the distribution of the data, the Friedman test was used to compare differences in adherence rates over time. A *P* value <.05 was considered to indicate statistical significance.

### Ethics Statement

The University of Technology Sydney Human Research Ethics Committee (HREC) approved this study (ETH19-3622). All users recruited into the program were required to actively accept and consent to the Terms of Use and Privacy Policy, which stated that de-identified data in aggregated form may be used by third parties for research and other purposes. No personal or confidential data were included in the database; therefore, informed patient consent was not required.

## Results

### Study Sample

A total of 130 users were included in the 6-month analysis, and 243 users were included in the 3-month analysis. For the timing adherence subanalysis, 111 users and 221 users were included in the 6-month and 3-month analyses, respectively.

#### 6-Month Analysis Group

The distribution of users according to gender was 36/130 male (27.7%) and 88/130 (67.7%); 6/130 users (4.6%) did not disclose their gender. The average age was 45.8 years (SD 17.2). The most common medications prescribed were rosuvastatin, cholecalciferol, and atorvastatin; the mean number of medications prescribed per patient was 4.3 (SD 3.1).

#### 3-Month Analysis Group

The distribution of users according to gender was 80/243 male (32.9%) and 156/243 female (64.2%); 7/243 users (2.9%) did not disclose their gender). The average age was 43.8 years (SD 15.5). The most common medications prescribed were varenicline, rosuvastatin, and cholecalciferol; the mean number of medications prescribed per patient was 4.0 (SD 2.9).

### Implementation Adherence

**Figure 1:**
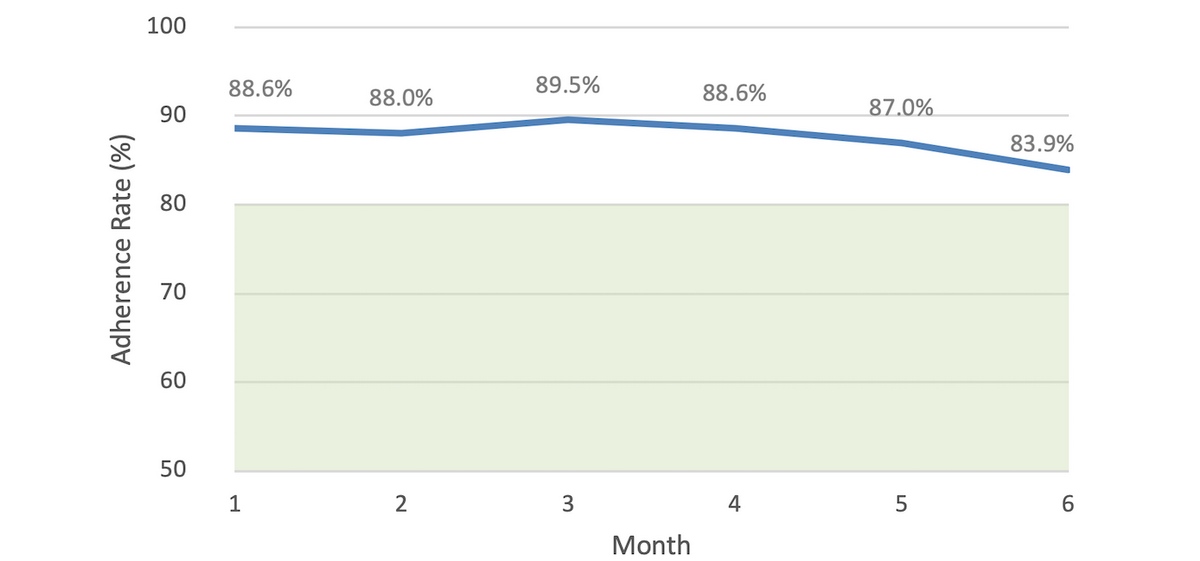
Mean implementation adherence rates of 130 users of the Perx app over 6 months. The shaded area below 80% indicates less than optimal adherence based on the literature.

Adherence rates across the 6-month time period are shown in Table 1. The overall median implementation adherence was 96.8% (IQR 87.1%-100%) across 6 months. A small decreasing trend was observed from month 4 to month 6. However, the Friedman test revealed non-significant differences in adherence rates over time (F_r_(2)=4.314, *P*=.505) (Figure 1).

**Figure 2:**
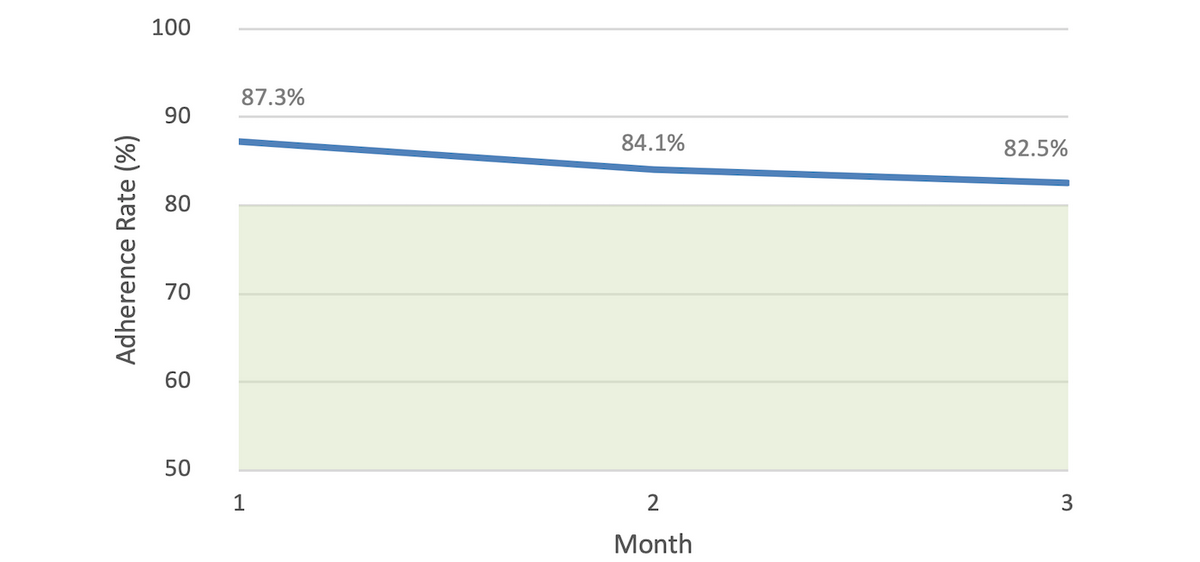
Mean implementation adherence rates of 243 users of the Perx app over 3 months. The shaded area below 80% indicates less than optimal adherence based on the literature.

Adherence rates across the 3-month time period are shown in Table 1. The overall median implementation adherence was 96.6% (IQR 82.1%-100%) across 3 months. A slight decreasing trend was seen from month 1 to month 3 (Figure 2). Similarly to the 6-month analysis, nonsignificant differences in adherence rates over time were found (F_r_(2)=0.635, *P*=.728).

**Table 1:** Adherence rates across the 6-month and 3-month time periods.

Study period		Mean (SD)	Median (IQR)
**6-month analysis (%)**			
	Month 1	88.6 (21.5)	96.8 (88.0-100)
	Month 2	88.0 (20.3)	96.8 (82.5-100)
	Month 3	89.5 (18.5)	97.1 (87.1-100)
	Month 4	88.6 (20.9)	98.3 (86.5-100)
	Month 5	87.0 (24.0)	97.1 (85.7-100)
	Month 6	83.9 (26.9)	96.8 (83.9-100)
	Overall	87.6 (16.9)	96.8 (87.1-100)
**3-month analysis (%)**			
	Month 1	87.3 (21.1)	96.1 (86.1-99.6)
	Month 2	84.1 (24.7)	96.8 (79.0-100)
	Month 3	82.5 (27.5)	96.7 (80.6-100)
	Overall	84.6 (20.9)	96.6 (82.1-100)

### Timing Adherence Subanalysis

**Figure 3:**
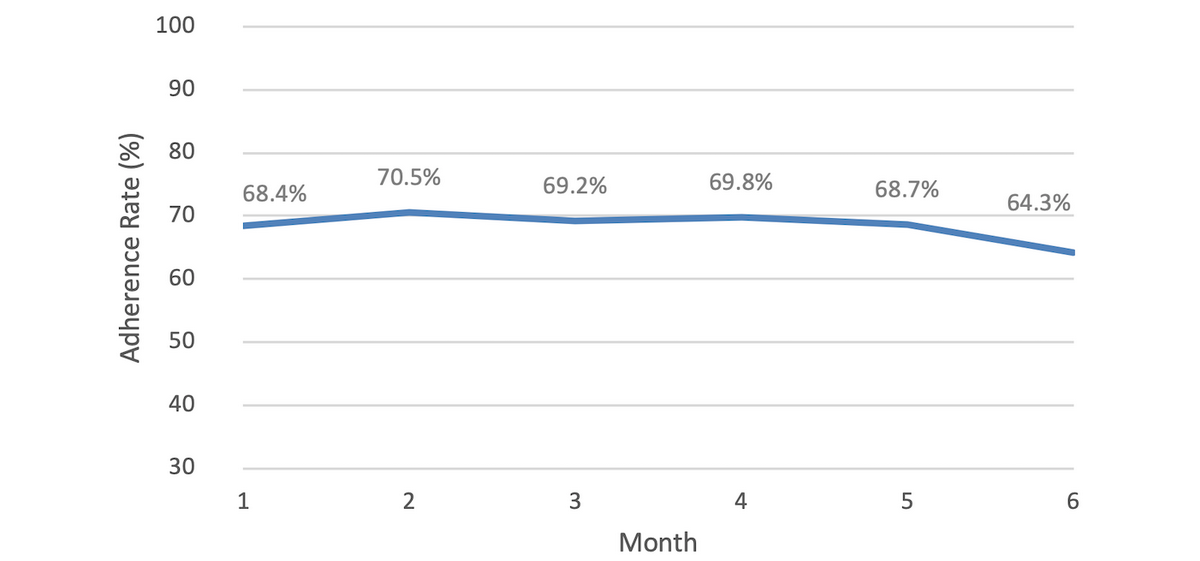
Mean timing adherence rates of 111 users of the Perx app over 6 months. The users were considered to be adherent to the dose if it was taken within ±1 hour of the scheduled time.

Timing adherence rates across study time periods can be found in Table 2. For the 111 users included in the 6-month timing adherence analysis, their adherence remained unchanged, with medians of 77.3% (IQR 52.0%-93.1%) in month 1 and 77.4% (IQR 36.2%-94.4%) in month 6. The median value across the time periods was 79.0% (IQR 50.8%-92.9%). Overall, there were no significant changes over time (F_r_(2)=5.465, *P*=.362) (Figure 3).

**Figure 4:**
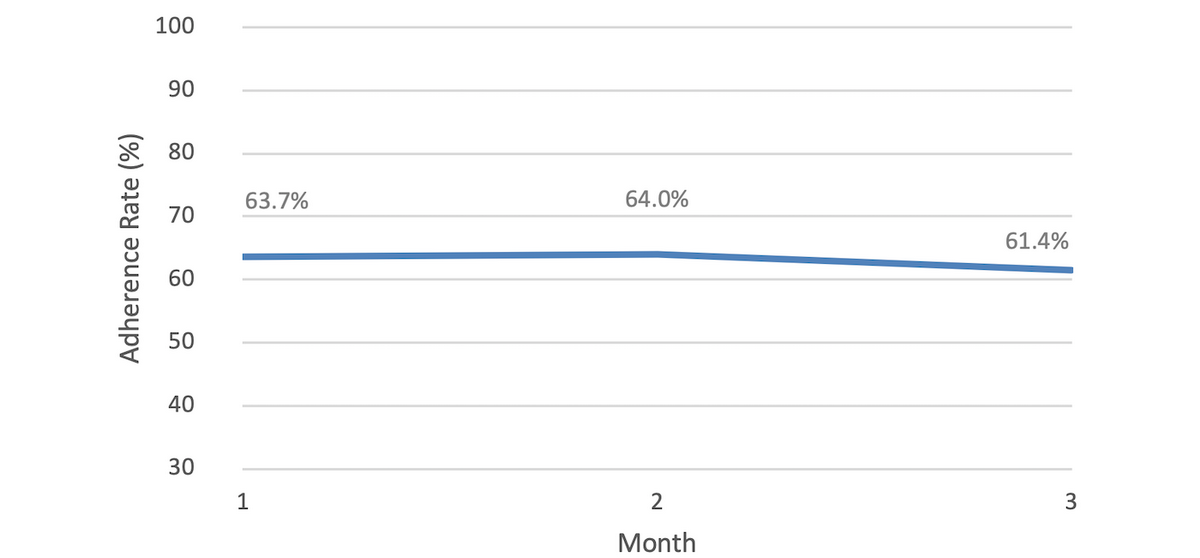
Mean timing adherence rates of 221 users of the Perx app over 3 months. The users were considered to be adherent to the dose if it was taken within ±1 hour of the scheduled time.

In the 3-month timing adherence analysis, 221 users’ adherence remained stable (Table 2), with nonsignificant changes across time periods (F_r_(2)=2.125, *P*=.346) (Figure 4).

**Table 2:** Timing adherence across the 6-month and 3-month time periods.

Study period		Mean (SD)	Median (IQR)
**6-month analysis (%)**			
	Month 1	68.4 (27.9)	77.3 (52.0-93.1)
	Month 2	70.5 (28.4)	82.3 (54.8-91.9)
	Month 3	69.2 (27.7)	79.8 (50.3-93.5)
	Month 4	69.8 (28.8)	81.7 (51.4-93.5)
	Month 5	68.7 (28.9)	80.6 (52.8-92.7)
	Month 6	63.4 (33.7)	77.4 (36.2-94.4)
	Overall	68.5 (29.1)	79.0 (50.8-92.9)
**3-month analysis (%)**			
	Month 1	63.7 (28.2)	71.0 (46.4-85.9)
	Month 2	64.0 (30.8)	74.2 (41.9-90.3)
	Month 3	61.4 (32.3)	71.0 (34.7-88.7)
	Overall	61.1 (28.5)	72.0 (41.8-88.3)

## Discussion

### Principal Findings

Retrospective analysis of the medication adherence of users receiving a multicomponent adherence management intervention that includes reminders, educational components, incentives, gamification, and social community components demonstrated that this intervention is a successful approach to maintaining optimal medication adherence. To our knowledge, this is the first study investigating a comprehensive multicomponent mobile intervention to maintain medication adherence across different chronic conditions.

Trends observed from the users of the mobile app showed high rates of adherence across the study periods. The adherence rates of Perx users averaged over 85% across six months. This was significantly higher than previously observed dispensing data adherence rates in Australian patients, which were found to be between 50.2% and 66.9% [38]. While a slight decrease in adherence was observed over 6 months, the long-term rates remained above 80%, which is often considered to be an optimal threshold for medication adherence [36]. The decrease in adherence rates was found to be statistically insignificant [36][39]. The gradual decrease was less pronounced than that in previous literature examining the long-term effects and multidimensional, dynamic nature of medication adherence; in a previous study, average adherence was estimated to decrease by 1.1% per month [40]. This suggests that the addition of gamification and incentive components to more traditional management interventions (eg, educational components and reminders) is a viable option to inspire long-term motivation and adherence to medications.

A recent network meta-analysis examined the impact of adherence interventions across time and identified multicomponent interventions as the most effective long-term solution [4]. Although there is limited evidence, interventions that include incentives and technical aspects (ie, dosage reminders) have been shown to be the most effective in sustaining long-term results [4]. The Perx digital therapeutic presents an advantageous alternative to existing medication adherence interventions due to its incorporation of multiple and innovative components into one platform to continuously motivate and empower users. A main component of the Perx app, medication reminders, has long been identified as a successful intervention component to improve adherence [41,42]. However, although medication reminders help to enhance adherence, they only affect one dimension of the multiple nonadherence determinants and are frequently used in combination with additional interventions, such as education [4]. Educational interventions are also a common long-term strategy to improve adherence to medications [2,43]. Delivered by numerous methods, these interventions can be moderately effective; however, they are not a sole solution to improve adherence for all patients [44]. When combined with technical and attitudinal components such as motivational interviewing, education-based strategies are found to be even more successful [4].

Motivation is another common determinant of medication adherence [3]. Patients can be fully aware of the positive health benefits medications provide as well as the consequences of poor health behavior; however, some patients consistently make poor health choices [11]. Present-biased preferences explain the “human tendency to grab immediate rewards and to avoid immediate costs in a way that our ‘long-run selves’ do not appreciate [10].” An individual may analyze immediate costs or immediate rewards to make a decision; such decisions often result from impatience or immediate gratification and place greater value on achieving gratification in the present moment than obtaining the same reward in the future. Positive and negative health outcomes remain too distant of a reward and consequence, respectively [45]. The Perx digital therapeutic aims to create instant gratification through gamification elements. Through receiving instant praise and reward after each medication dose taken on time, users may be motivated to continue to be adherent. Motivation can additionally be created through intrinsic forces, as stated by the self-determination theory. The self-determination theory suggests that the nature of perceptible motivational types determines the predictability and force of how people behave, rather than the amount of motivation [46,47]. Therefore, it is necessary for gamified systems to promote a sense of autonomy, competence, and relatedness to create the intrinsic motivation needed to continue the value of the extrinsic motivating factors [46,48]. The Perx digital therapeutic intervention may be successful because it meets the users’ need for competence, autonomy, and relatedness. Competence and autonomy are created by setting challenging yet manageable goals, where adherence is the challenge and financial incentives are the goals. Users can also follow their progress through points, leaderboards, and personal visualized feedback graphs on their individual adherence. This feedback provides additional positive reinforcement and has been proven to be a successful component of interventions to improve medication adherence; it is estimated that adherence increases 8.8% for interventions where feedback is included compared to those that do not include feedback [40,49]. The social community component meets the need for relatedness by fostering a feeling of belonging to a community that shares the common goal of better health [46].

While gamification is a main force in creating motivation in the app, the impact of rewards and incentives cannot be dismissed. The use of incentives in public policy has long been used as an extrinsic force to influence behavior and intrinsic motivation [9,50,51]. However, the use of incentives to encourage health behaviors is relatively new, and more research is needed in this area. Financial incentives have proven to improve medication adherence in certain populations; however, their long-term viability can be questioned due to the resources needed [13-19]. Although incentives can be critiqued on their superficial nature or short-term viability, they may be a powerful motivating factor in creating habit-based behavior, a proven successful key in improving medication adherence, and an intrinsic source of motivation [51,52]. In the case of Perx, the extrinsic nature of the incentives may create habit-based adherence behavior in addition to intrinsic motivation to improve health outcomes. Additionally, the Perx app uses lottery-based incentives rather than predictable rewards. These incentives can enhance health behavior based on regret aversion or the human tendency to place a significant cost on regret [20]. If users believe that missing a medication dose can prevent them from winning a reward, they are still likely to improve their adherence, even without a guaranteed instant reward [21].

### Limitations

Although our analysis proved that the Perx digital therapeutic is an effective intervention in managing medication adherence, it does have some limitations. First, the number of app users with available data was limited, did not extend past 6 months for the majority of users, and did not include information on the users’ clinical conditions. Due to this, we were unable to perform subanalyses based on patient age, gender, medication, or condition. Second, we could not establish baseline adherence rates before the intervention was implemented or evaluate a control group due to the retrospective nature of the study. Third, while we believe that our sample reflects an accurate sample of patients who would be likely to use a mobile app to manage medications, the users who downloaded the app may also have been likely to adhere to their medications without the app. Conversely, it could also be argued that patients who need adherence management support would be more likely to download the app. Finally, while we could measure the number of active days per patient, it was not possible to determine full user engagement of the intervention in this analysis to understand the extent to which the intervention was used by each user.

### Strengths

One strength of our study is our measure of adherence, self-reporting with MDOT [35]. Similar to electronic methods such as the Medication Event Monitoring System (MEMS), MDOT enables objective measurement while simultaneously providing timestamps to additionally measure timing adherence, which is an important component of the multidimensional medication-taking process [35]. Additionally, our analysis of the gamification of mobile apps to maintain positive health behaviors is part of a new and emerging research landscape within the pharmacy and health care sector that has not been previously examined [53]. With the increasing number of health apps entering the market, supportive evidence is necessary to demonstrate the effectiveness of these tools and to indicate whether they should be recommended by health care professionals as a component of medication therapy [54,55]. Finally, our use of real-world data generated from users of this commercially available mobile app was a strength in that the data can be applied to a broader population of patients and reflect actual use in practice [32].

### Future Work

A 12-month clinical trial is currently being conducted with the objective of assessing the efficacy of the Perx intervention in adherence and clinical outcomes. Future research should aim to assess the effectiveness of this intervention in improving adherence to medications and other gamification- or incentive-based strategies in addition to observing the impact on clinical health outcomes. Furthermore, a longer analysis period of 12 to 24 months would be beneficial in observing the long-term effects to determine if these types of interventions can sustain gold-standard adherence rates above 80% for longer than 6 months. It would additionally be useful to analyze the impact of the intervention across different points in the medication-taking process, such as initiation of medication, implementation and persistence adherence, and time to discontinuation of medication [1]. Finally, the opinions of stakeholders, specifically users, regarding the app and intervention components are vital to understand the main motivating factor in promoting adherence. A full engagement analysis identifying the components of the app on which the most time is spent as well as a user survey analysis are required to obtain a complete understanding of the success of the intervention.

### Conclusion

Retrospective analysis of a digital therapeutic mobile app that merges gamification, education, reminders, a social community, and incentive-based components indicates that this intervention is successful in maintaining optimal medication adherence over time. Extrinsic external monetary motivators combined with fundamental game mechanics and other common behavioral change components may be a key force to promote intrinsic motivation and habit-based behavior, which can spark long-term changes in health behavior. Future research should evaluate the long-term impact of mobile apps using these components over a longer time period using experimental designs.

## Appendix

Multimedia Appendix 1 Screenshots of the most important features of the Perx app.

## Abbreviations

EMERGE: ESPACOMP Medication Adherence Reporting Guideline
MDOT: mobile direct observation of therapy
MEMS: Medication Event Monitoring System
mHealth: mobile health
STROBE: STrengthening the Reporting of OBservational Studies in Epidemiology
