# Supplementary figures and images for: Impact of a Multicomponent Digital Therapeutic Mobile App on Medication Adherence in Patients with Chronic Conditions: Retrospective Analysis
Source: J Med Internet Res. 2020 Aug 12;22(8):e17834. doi: 10.2196/17834 (PMC7450368; doi:10.2196/17834)

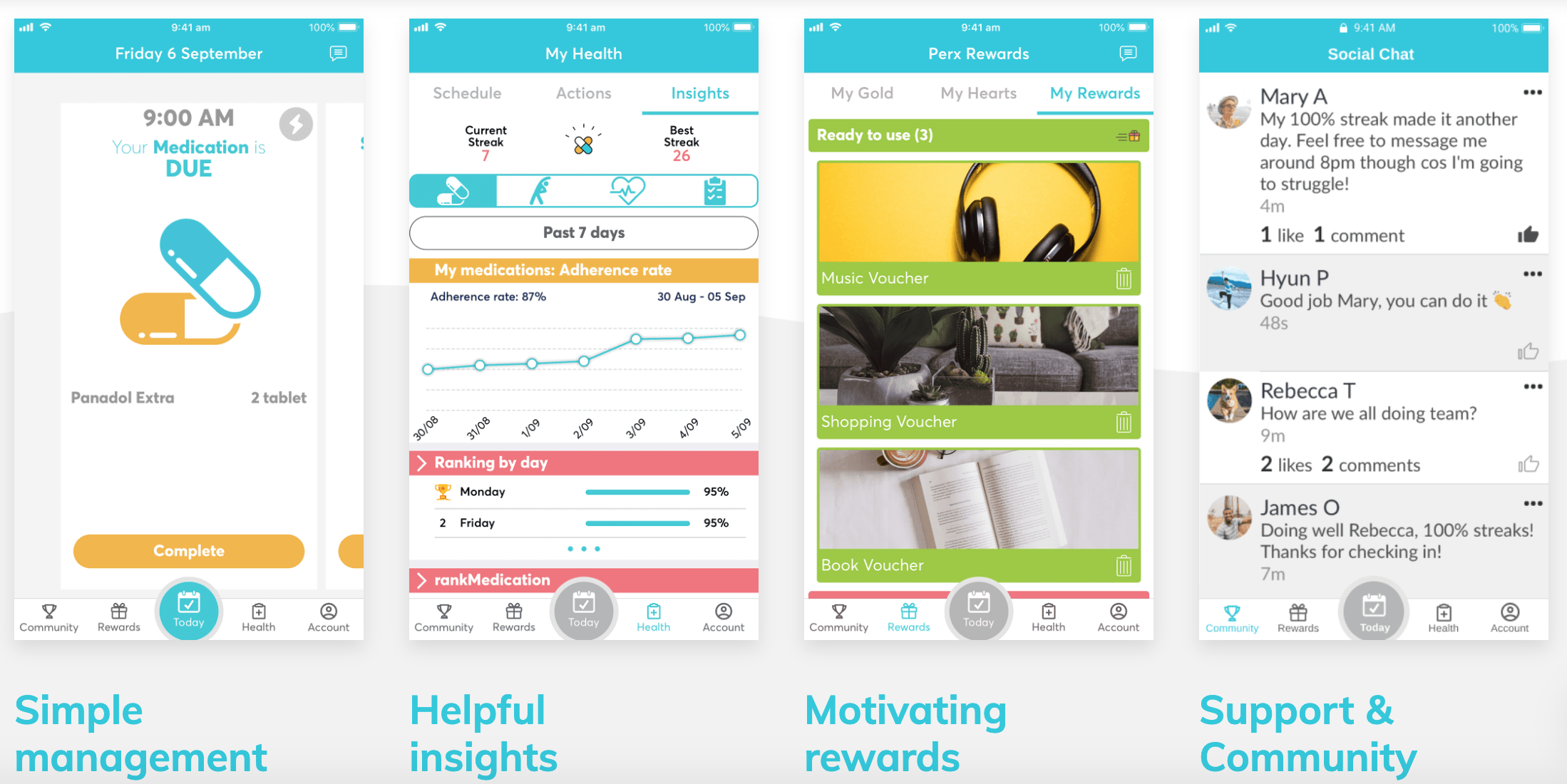

Supplement: Multimedia Appendix 1 [file jmir_v22i8e17834_app1.png]
